# Pregnancy after Uterine Artery Embolization: A Case Report in a Woman with Leiomyomata

**DOI:** 10.1155/2015/235312

**Published:** 2015-01-29

**Authors:** Helena Isabel Lopes, Maria Isabel Sá, Rosa Maria Rodrigues

**Affiliations:** Department of Obstetrics and Gynecology, Centro Hospitalar do Porto, 4099-001 Porto, Portugal

## Abstract

*Background*. Several pregnancies have been reported after embolization of uterine artery. This procedure is an accepted nonsurgical treatment for symptomatic uterine fibroids but its safety in women desiring future childbearing is not well established. *Case Report*. We present a 40-year-old woman with leiomyomata who became pregnant after previously undergone uterine artery embolization for three times. The placenta was previa and the fetus was in transverse position. She had a cesarean delivery of an appropriately grown fetus at 37 weeks, which was followed by uterine atony requiring hysterectomy. *Conclusion*. Although pregnancy-related outcomes remain understudied, the available reports evidence that pregnancies after uterine artery embolization may be at significantly increased risk for postpartum hemorrhage, cesarean delivery, abnormal placentation, and malpresentation. In patients who are undergoing this type of treatment and contemplating pregnancy, the possibility of adverse complications should be taken in consideration and women should be appropriately advised.

## 1. Introduction

Uterine fibroids are one of the most common benign tumors, with a prevalence of 30% among women of reproductive age. Although most women are asymptomatic, there are others that experience bothersome bulk-related symptoms like pelvic pain, pelvic pressure, urinary frequency, and abnormal uterine bleeding [1]. Uterine artery embolization (UAE) is a nonsurgical treatment option for these women and who wish to retain their uterus and avoid surgery [2]. Recommendation of UAE in women desiring pregnancy is controversial since there are concerns about its effects on fertility and pregnancy [3].

This paper reports a case of pregnancy and its outcomes in a woman who had previously submitted to three uterine artery embolizations for symptomatic leiomyomata.

## 2. Case Presentation

We present a case of a 40-year-old woman, gravida 4 para 1, with antenatal surveillance of her pregnancy in our institution. Her first pregnancy and labor occurred normally, fourteen years ago. Some years later, she started experience dysmenorrhea, pelvic pain, and heavy menstrual bleeding. The diagnosis of leiomyomata was made by ultrasonography and magnetic resonance imaging (MRI), showing a uterine volume of 580 cc with a dominant intramural fibroid of 105 cc volume in the anterior wall. She suffered two miscarriages after this diagnosis. After counseling about her different options of treatment, she always refused any type of surgery, trying to preserve her uterus and expecting a future pregnancy. Then, uterine artery embolization was performed with decrease in fibroid size of 24% after 6 months and improvement of symptoms. However, one year after, the symptoms recurred and uterine myomas returned to a previous size. She undergone for more 2 UAE in another center. The procedure was repeated in the second time because only 30% degree of fibroid ischemia was achieved after the first embolization. Moreover, MRI revealed some contributing circulation from the left ovarian artery.

**Figure 1:**
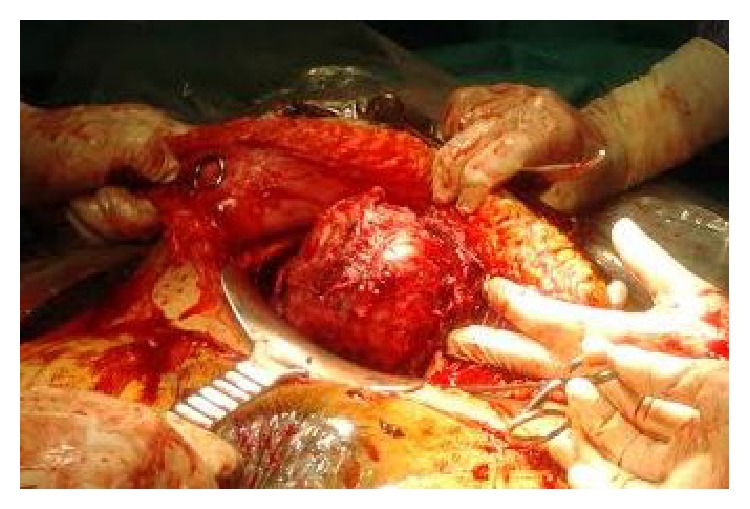
Large intramural leiomyoma protruding from the anterior lower segment (after dissection of anterior pseudocapsule).

Three months after embolization she became pregnant spontaneously. On antenatal ultrasound scans several uterine intramural fibroids were evident including the dominant one placed at the lower segment of anterior wall measuring about 65 × 60 mm. The placenta was previa and the fetus was at transverse situation. For these reasons and because of vaginal bleeding incident, a cesarean section was performed at 37 weeks of gestation. Intraoperatively the lower segment was thickened due to underneath leiomyoma. A longitudinal hysterotomy was needed to access the amniotic sac. A 2970 g female fetus was delivered, with Apgar scores of 5 and 8 at 1 and 5 minutes, respectively. The placenta was delivered manually. After uterine contraction was noted, the prominence of the large leiomyoma with about 10 cm size in the anterior lower segment (Figure 1) and respective myomectomy was performed. A prophylactic Bakri balloon was left inside of endometrial cavity prior to complete hysterorrhaphy in three-layer suture. The remaining uterus was increased in size due to the presence of other smaller intramural leiomyomas, but its tonus was consistent.

**Figure 2:**
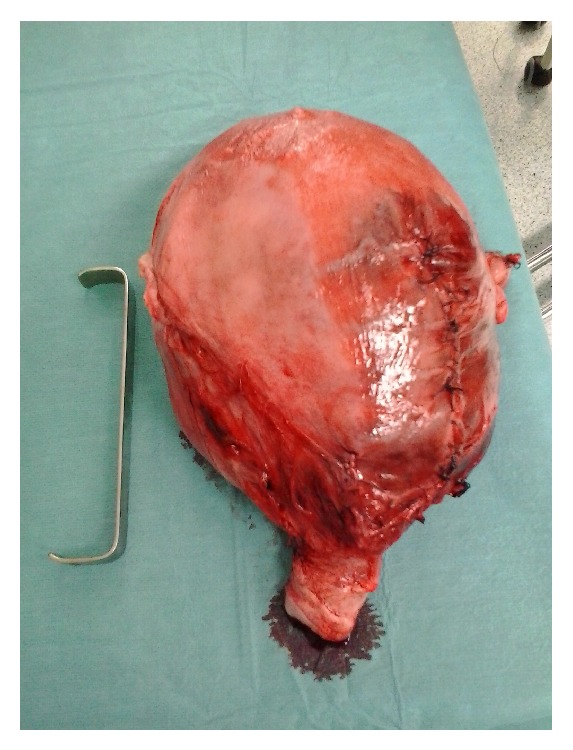
Uterus after postcesarean hysterectomy (median suture of hysterorrhaphy is seen).

Two hours later on postoperative recovery room, she developed atonic hemorrhage and became hemodynamically instable. A total hysterectomy was performed as a life-saving measure (Figure 2). During surgery the patient was transfused with red blood cells, fresh frozen plasma, and platelets, in order to control the disseminated intravascular coagulopathy. Histopathology of the uterus showed adenomyosis, interstitial and subserous leiomyomas, and signs of previous embolization. The placenta showed no histopathological alterations.

The patient recovered well postoperatively and was discharged home on postoperative day 12.

## 3. Discussion

Uterine artery embolization was first described by Ravina in 1995 and has been shown to be an effective alternative treatment for symptomatic uterine leiomyomata. Outcomes data regarding women who desire future fertility are less clear and limited [4]. This case demonstrates that pregnancy after embolization is possible and can occur soon after the procedure. McLucas showed recently that pregnancy is a viable option for women undergoing UAE. In his review, the total pregnancy rate (pregnancies achieved amongst women under 40 who desired pregnancy) was 63.4% and 48% having successful term pregnancies [5].

The safety of pregnancy has not been established either. Several case series of pregnancies following UAE have reported some adverse outcomes. Our patient index had a pregnancy complicated by placenta previa, fetal malpresentation, cesarean delivery, and postpartum hemorrhage. Walker and McDowell in a series of 56 completed pregnancies after UAE for fibroids have reported placenta previa rate of 14.3%, cesarean rate of 72.7%, and postpartum hemorrhage rate of 18% [6]. A large multicentric trial from Ontario reported abnormal placentation in 12.5% of cases [1]. Another series of 16 viable pregnancies among 671 women underwent UAE for fibroids and reported a cesarean rate of 88% and postpartum hemorrhage rate of 18% [7]. Goldberg et al. in 2002 reviewed all pregnancy reports to date in addition to their 2 additional cases. Among 23 pregnant women whose indication for embolization was symptomatic leiomyomata, there was a 22% rate of malpresentation, 65% cesarean delivery rate, and a 9% rate of postpartum hemorrhage [4]. Theoretically, the devascularization of the myometrium resulting from the embolization procedure can affect its ability to successfully contract following delivery [4]. On the other hand, there is a high association between leiomyomata and postpartum hemorrhage [8]. In our case, the presence of residual leiomyomas was certainly a major contributing factor to uterine atony and postpartum hemorrhage requiring hysterectomy, despite prior embolization. In the same way, it confers a similar risk to placenta previa, fetal malpresentation and cesarean delivery [8]. Homer and Saridogan meta-analyzed 227 women with fibroids that achieved pregnancy after UAE. The cesarean section rate (66%) and postpartum hemorrhage rate (13.9%) were statistical significantly higher in post-UAE pregnancies than in fibroid-containing pregnancies (48.5% and 2.5%, resp.). Rate of malpresentation was similar in both groups [9].

The presence of ovarian-uterine anastomoses that provide collateral blood flow to the uterus (showed by MRI after the procedure) may have been the cause of partial failure of UAE and thus the persistence of residual leiomyomas [10], showing an unsuccessful UAE, giving the patient an increased risk.

Women who are undergoing this type of treatment and contemplating pregnancy must be adequately informed about potential complications. If residual uterine fibroids persist, it may influence the main obstetric outcomes, regardless of the potential effect of embolization on pregnancy.
